# Community wellbeing and lived experiences during urban redevelopment in New Zealand: Te Hotonga Hapori – Connecting communities qualitative study protocol

**DOI:** 10.1371/journal.pone.0333480

**Published:** 2025-10-03

**Authors:** Erica Hinckson, Vivienne Ivory, Julia McPhee, Megan Somerville Ryan, Moushumi Chaudhury, Lisa Mackay, Albert Refiti, Abby C. King, Tania Ka’ai, Scott Duncan

**Affiliations:** 1 Human Potential Centre, School of Sport, Exercise and Health, Faculty of Health and Environmental Sciences, Auckland University of Technology, Auckland, New Zealand; 2 WSP Research & Innovation, Wellington, New Zealand; 3 Vā Moana Research Cluster, Art and Design, Faculty of Design and Creative Technologies, Auckland University of Technology, Auckland, New Zealand; 4 Department of Epidemiology & Population Health, Stanford University School of Medicine, Stanford, Palo Alto, United States of America; 5 Stanford Prevention Research Center, Department of Medicine, Stanford University School of Medicine, Stanford, Palo Alto, United States of America; 6 Te Ipukarea Research Institute, Auckland University of Technology, Auckland, New Zealand; Universitat de Girona, SPAIN

## Abstract

Cities around the world are growing rapidly, and the demand for housing is subsequently increasing. Many governments have initiated large-scale redevelopment projects to address the problem but planning and implementation can significantly impact the wellbeing of residents. Evidence has shown that people-centred urban planning and redevelopment, including walkable areas, natural environments, and appealing public spaces, can enhance physical, mental, and social wellbeing. The impact of these designs on wellbeing is complex, and without community involvement, there is a risk that redevelopment may not meet the wellbeing needs of the residents. Community Wellbeing and Lived Experiences study is part of Te Hotonga Hapori – Connecting Communities research programme which aims to provide the evidence to improve liveability and wellbeing in neighbourhoods that are undergoing redevelopment. It seeks to empower communities by involving them directly in the redevelopment process. Guided by the culturally informed Te Hotonga Hapori Engagement Framework it comprises 5 components: i) Active relationship building; ii) Historical/cultural realities; iii) Community aspirations; iv) Building bridges; and v) Activation of neighbourhood urban and natural environments. Data collection will be undertaken using the Community Science Aotearoa process that is contextually specific to communities in New Zealand and adapted from the *Our Voice* Citizen Science Research method. Residents using Te Hotonga Hapori app will collect photos and narratives and share information about their lived experiences during the redevelopment using a six-step process of Engage, Discover, Discuss, Advocate, Change and Re-engage. Descriptive statistics will be produced in relation to the number and typology of photos and narratives collected. Qualitative thematic analysis will be used for narrative data obtained from downloaded app data, and community and group sessions. Braun and Clarke’s Thematic Analysis Framework will guide researchers through an iterative process and themes will be developed using the Attride-Stirling Thematic Networks Analytic Tool.

## Introduction

Cities are projected to hold close to 5 billion people by 2030 [1]. Alongside the massive growth is the demand for housing, which drives up house prices and presents issues of house affordability. While governments may respond by funding large redevelopment projects, how these redevelopments are planned, designed and executed can impact significantly individual, family and community wellbeing [2]. We define community wellbeing as the collective social, cultural, environmental, and relational conditions that enable individuals and groups to thrive in ways that are equitable and sustainable. A central component of wellbeing is the opportunity for community interaction.

There is an opportunity for neighbourhoods that are designed and planned according to evidence [3] to enhance wellbeing of the residents by enabling a streetscape that supports active living. For example, infrastructure that enables convenient and safe active travel, quality public transit and consequently less reliance on car use [4], designing public spaces for wellbeing, such as community sensory gardens [5], and generally integrating nature into design such as parks and lakes [6]. There is evidence to show that compact neighbourhoods with mix uses [7], bring people together to create vibrant communities [8], increase opportunities for physical activity [9], reduce isolation and loneliness [10], and in general increase social cohesion [11]. While we acknowledge that community interaction is not confined to geography and may take shape through professional, cultural, or online networks, neighbourhoods remain critical sites where everyday interactions unfold. They are sites where redevelopment interventions occur and where built environments most directly shape opportunities for connection, safety, mobility, and access to resources. We see neighbourhoods as focal points or “natural” hubs of community life, for any intervention where the lived experiences of residents and the material conditions of place intersect most directly [2].

However, without community involvement and participation during planning and subsequent implementation, there is a serious risk of developing neighbourhoods that are not fit for their intended purpose, resulting in several irreversible challenges, particularly social, economic and environmental. We use the term “community” to refer both to collective groups and to the individuals and whānau (extended family) who reside within a neighbourhood. We acknowledge that communities are dynamic and may include long-term residents, newly arrived households, and those who have experienced displacement. Community participation is therefore understood as flexible and multi-scalar, encompassing contributions at the individual, whānau, and collective levels.

While cautionary examples such as Pruitt-Igoe, the Heygate Estate, and the West Kowloon Cultural District [12–15] are often cited as illustrative failures of top-down redevelopment, their histories are more complex than design alone. Pruitt-Igoe’s demolition, for instance, has too often been attributed solely to its modernist architecture, whereas evidence points to intersecting factors including underfunded maintenance, poor management, racial segregation, and broader economic decline [12,13]. Similarly, the Heygate Estate involved not only physical design issues [14] but also large-scale displacement of residents and the erosion of affordable housing, [16] while the West Kowloon Cultural District has been critiqued for governance practices that marginalised community voices and prioritised commercial interests [15]. These examples highlight that redevelopment approaches fail not simply because of their physical form or professional determinism, but because they pre-determine outcomes and processes without early and meaningful engagement with those most affected. Even apparently participatory programmes, such as HOPE VI in the United States, have been critiqued for displacing residents and reducing affordable housing despite their promotion of more ‘traditional’ walkable neighbourhoods [17]. Together, these cases demonstrate the risks of redevelopment processes that engage communities superficially, symbolically, or too late, and reinforce the importance of authentic, sustained participation in shaping urban transformation.

Citizen and community science can be used when proposed changes are directly affecting communities [18]. The science component typically is based upon robust research frameworks from which to share learnings and evidence on the most effective community participation methods. This type of scientific endeavour draws directly from the untapped potential of community members and gives voice to the voiceless. It has the potential to build capacity within the community while at the same time enhancing social cohesion. Citizen Science can be defined as an approach focused on involving members of the community working closely with research investigators to advance research projects [19]. Aligned with community-based participatory research (CBPR), it has the potential to impact society and social infrastucture in a direct and meaningful way. Integrating citizen science with a participatory action approach that includes community residents in the fuller scientific process – called the *Our Voice* Citizen Science for Health Equity “by the people” method [20] – helps empower participants to provide signficant and meaningful local information and perspectives about their environment. This method allows participants to prioritise their concerns, create their own interpretations of the data, and engage in cross-sector conversations to generate practical solutions that have impacts on their neighbourbood. This type of method is similar to some Community Science approaches which are led or co-led by communities themselves, focusing on addressing specific issues or challenges that directly impact them [21]. Community members partner with researchers and relevant community stakeholders, to address social or environmental change. The outcomes of community science projects are typically more applied than other forms of science, aiming to produce tangible benefits for the community, such as policy changes, improved health outcomes, or environmental improvements. Engaging with and empowering communities early to identify challenges and solutions is a route to improved redevelopment projects while at same time addressing residents’ wellbeing [22]. There is however less research on how communities can be empowered during extended development and construction in their neighbourhoods.

In this study, we are interested in understanding the diversity of experience at the people-centred level during neighbourhood redevelopments and offer a pathway of empowerment by engaging residents to collect and then apply their own data. We hypothesise that by applying a contextually specific community science participatory process that empowers communities, residents will be given the voice, skills and process to explore their experiences in depth, communicate these to stakeholders involved in the redevelopment process and champion change for their neighbourhoods. In addition, we seek to empower residents to be able to continue advocating for the community post study implementation.

The aim of this study is to empower communities to have a say about the urban redevelopment of their communities. This is guided by Te Hotonga Hapori Engagement Framework, which embeds Māori and Pacific principles into participatory practice, and operationalised by the Community Science Aotearoa process, an adaptation of the internationally recognised *Our Voice* citizen science for health equity method. Together, these approaches provide both the guiding principles and the iterative processes through which community perspectives can be meaningfully integrated into urban development decisions in Aotearoa New Zealand.

Our framing is grounded in the recognition that redevelopment processes have too often been dominated by professional expertise and technical determinism, frequently at the expense of community knowledge [12,13]. By centring diverse lived experiences, we take the explicit position that communities hold forms of expertise that are essential to equitable and sustainable urban transformation. We acknowledge that this position rests on assumptions that require critical reflection. Participation is not inherently positive: without depth, reciprocity, and cultural grounding, it risks becoming symbolic or extractive [23]. Likewise, ideals of “community” and “neighbourhood” are contested, shaped by histories of redevelopment that have displaced, fragmented, or redefined local identities. Situating this study and its protocol within broader debates about expertise, participation, and neighbourhood form shows both the importance and the complexity of placing lived experience at the heart of urban redevelopment. This positioning provides the rationale for developing and testing an approach that foregrounds people’s voices while critically engaging with the limitations and challenges of participatory practice.

We adopt the concept of a “mosaic of evidence” to emphasise that robust urban health and wellbeing solutions require multiple, complementary sources of knowledge [24–26]. Within this mosaic, Te Hotonga Hapori Engagement Framework and Community Science Aotearoa Process will contribute the lived experience and cultural perspectives that are often absent from conventional planning data. These insights can sit alongside epidemiological evidence, urban planning metrics, and policy analysis, creating a richer and more contextualised evidence base.

The study sits within Te Hotonga Hapori – Connecting Communities research programme, addressing the goals of improving liveability and community wellbeing.

## Materials and methods

We used the standardised checklist “Standards for Reporting Qualitative Research” (SRQR) recommendations. [27] SRQR reporting is utilised to ensure transparency and report on all aspects of the qualitative research.

### Design

Te Hotonga Hapori-Connecting Communities Research Programme seeks to investigate how urban redevelopment planning and delivery can be optimised to enhance neighbourhood liveability and community wellbeing through a series of five interconnected projects. In this paper, we describe the methods of the *Community Wellbeing and Lived Experiences* project which employs a qualitative, community based participatory research method. The study will be located in the North Island of Aotearoa New Zealand, in the city of Tāmaki Makaurau Auckland, the largest city in New Zealand with a population of over 1.5 million residents (one-third of the country’s population) [28]. Data will be collected in four areas undergoing redevelopment: Oranga (population, 3198), Aorere (pop, 8718), Wesley (pop, 5652), and Waikowhai (pop, 5439) [28] during 2024–2025. Site selection was guided by several criteria: (i) the scale and extent of planned redevelopment, ensuring that the sites represent substantial urban redevelopment with potential impacts on wellbeing; (ii) demographic diversity, including high proportions of Māori, Pacific, and migrant communities, and a range of socioeconomic contexts; (iii) variation in redevelopment stage, with sites spanning early planning through to active construction, allowing the framework to be tested at different phases of redevelopment; and (iv) feasibility considerations, including existing partnerships with local councils, health promotion agencies, and community organisations, which provide culturally safe and trusted entry points.

### Participants, sampling and recruitment

Recruitment for Te Hotonga Hapori research programme will be conducted by a team of interviewers with extensive experience in Door-to-Door methodology. The interviewers will systematically approach all eligible households in each study area, making up to four return visits at varying times of the day and across different days of the week to ensure maximum engagement. In each household, one adult will be recruited using the ‘next birthday’ sampling method. During the consent process participants will be asked if they would be willing to be recontacted for potential participation in the current study-Community Wellbeing and Lived Experiences study. Those who consent to further contact will then be considered as prospective participants.

Residents in the four study areas who participated in the earlier Te Hotonga Hapori projects (AUTEC 21/361) and consented to be contacted for this project will be invited to participate. An invitation letter will be sent with the participant information sheet detailing the study and its requirements along with a consent form. Potential participants will be given two weeks to consider the invitation to provide their written informed consent. Participants will include residents housed in publicly owned and private dwellings. We will invite up to 30 participants from each study area, for a total of approximately 120 participants aged 18 years and over. Participants will be offered a $50 voucher at three time points during the Community Science Aotearoa process (see below) as a thank you for their participation. Recruitment begun on the 28^th^ of March 2024 and it is ongoing. It is estimated that participant recruitment, data collection and results to be completed April 2025, July 2025 and October 2025 respectively. 

### Te Hotonga Hapori engagement framework and community science aotearoa process

In this section, we describe Te Hotonga Hapori Engagement Framework that will guide the implementation of the Community Science Aotearoa Process. Te Hotonga Hapori Engagement Framework provides an overarching, culturally grounded foundation for participatory engagement in Aotearoa New Zealand. It sets out five guiding phases, **Active** **Relationship Building, Historical and Cultural Realities, Community Aspirations, Building Bridges, and Activation of Neighbourhood Environments**, that define the principles underpinning engagement. Indigenous community experts from social and health promotion agencies in Auckland, New Zealand were engaged early. Through a series of sessions and applying content analysis methods, Te Hotonga Hapori Engagement Framework was developed that incorporates and reflects Māori and Pacific cultural perspectives, [29]. The Framework sets the foundation for the Community Science Aotearoa process which is based on the Our Voice Citizen Science for Healthy Equity (Our Voice) method and represents the adapted six-step process of **Engage, Discover, Discuss, Advocate, Change, Re-engage** that operationalises Te Hotonga Hapori Engagement Framework in practice. In other words, Te Hotonga Hapori Engagement Framework provides the guiding principles, while Community Science Aotearoa process provides the iterative process through which these principles are enacted. The *Our Voice* method, which includes the four basic steps of Discover, Discuss, Activate, Change [20] was developed by Professor Abby King and colleagues at Stanford University School of Medicine, USA [19]. This method has been designed to be readily adapted to the contexts and needs of diverse communities around the world [30,31]. In the current application of this method, the process of data collection and other steps were contextualised for Aotearoa, New Zealand residents [29]. See below for more details.

### The framework

**Fig 1:**
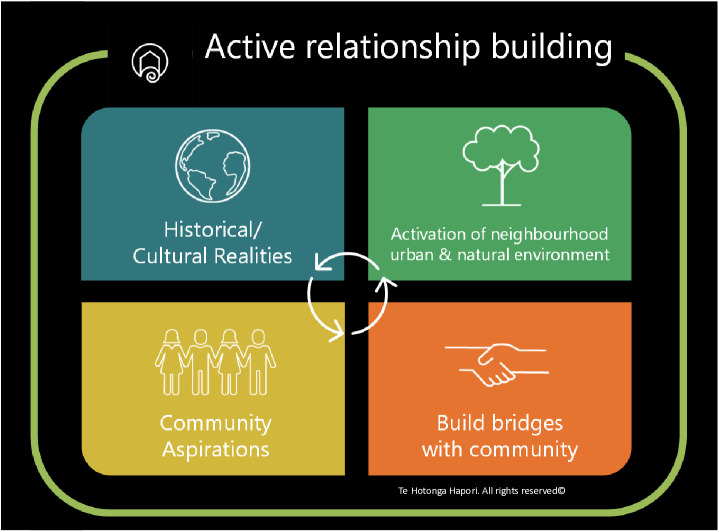
Te Hotonga Hapori Engagement Framework©.

Te Hotonga Hapori Engagement Framework incorporates and reflects Māori and Pacific cultural perspectives, therefore giving rise to a novel approach that is congruent with the unique ways of being within these indigenous communities. These cultural perspectives were gained through a series of collaborative sessions with representatives from local health promotion agencies that offered important insights and perspectives. The sessions were instrumental in ensuring the development of a framework that was culturally relevant and deeply rooted in the lived experiences and values of Māori and Pacific people. The framework is guided by Te Tiriti o Waitangi (Treaty of Waitangi) and emphasises the value of relationships, equity, sovereignty, governance and spirituality to ensure respect and empowerment of Indigenous communities. On that basis the framework includes five phases that interact in a cyclical manner: Phase 1. Active relationship building which extends through phases 2–5; Phase 2. Historical and cultural realities, Phase 3. Community aspirations, Phase 4. Building bridges, and Phase 5. Activation of neighbourhood urban and natural environments. See below for details (Fig 1).

#### Phase 1: Active relationship building.

The first phase is about establishing nurturing and sustaining relationships throughout the project. This means proactive, regular and authentic engagement with communities and relevant stakeholders to establish connection, collaboration, and reciprocal understanding. This aligns with extensive evidence from Community-Based Participatory Research (CBPR), which identifies trust and mutual benefit as prerequisites for authentic engagement [32]. Within the Aotearoa context, this principle is further supported by Kaupapa Māori research (research that centres on Māori self-determination), which positions whanaungatanga (the building and maintenance of relationships) as fundamental to ethical practice [23,33]. The first phase encapsulates all other phases in that it is fundamental, ongoing, and continuous, bringing to the forefront the importance of relationships. Relationship building is not assumed to be a one-off stage, but an iterative and ongoing process throughout the framework. Lessons from past participatory approaches highlight that failure to invest in relationships, treating them as procedural rather than relational, can lead to mistrust, disengagement, and tokenistic outcomes [34]. Our framework therefore emphasises mana-enhancing interactions (uplifting) that prioritise reciprocity and care, creating conditions in which communities can safely and meaningfully contribute to redevelopment processes.

The purpose of this phase is to establish and keep channels of communication open to ensure that the needs of the community are met, and concerns are identified early and addressed in a timely and proactive manner. Throughout this phase, the research team will proactively connect with communities undergoing redevelopment through community events, festivals, and market days. It will participate in all invitations to present the proposed research to local boards, local community groups, iwi (Māori tribes) and other cultural groups or local businesses. Community leaders will be identified through existing networks, recommendations from community members, and advice from Māori and Pacific cultural advisors or groups, acknowledging that leadership may be formal (e.g., organisational representatives) or informal (e.g., respected community members who hold influence through lived experience). Initial contact will be made through meetings in person or online as required, guided by protocols of Whakawhanaungatanga (relationship building through the sharing of connections), to establish trust and clarify the purpose of engagement. As the project progresses, decisions about who to engage will therefore be iterative and informed by continuous dialogue with community partners, ensuring that relationships remain authentic, inclusive, and responsive to community dynamics. This phase ensures an inclusive and participatory approach before the project begins.

#### Phase 2: Historical and cultural realities.

In the second phase, understanding the social and cultural realities that affect communities is the critical component. It goes beyond simply acknowledging the current social and other circumstances of the area and it necessitates an in-depth exploration of the history of the place to understand how and what has shaped the community over the years. It recognises that urban redevelopment is not a neutral process but is embedded within histories of colonisation, dispossession, and cultural erasure. Indigenous planning scholarship emphasises that acknowledging these histories is essential for building legitimacy and avoiding the reproduction of inequities [35,36]. In Aotearoa New Zealand, Māori scholars have argued that planning processes which disregard Te Tiriti o Waitangi and local cultural narratives risk perpetuating structural injustices [37]. Internationally, similar critiques highlight that redevelopment projects which fail to engage with cultural histories can fracture community identity and trust [38]. At the same time, recognition alone is insufficient: lessons from past projects show that symbolic acknowledgements, without material change, can exacerbate mistrust [39]. Te Hotonga Hapori framework therefore situates redevelopment within both historical and cultural realities, creating space for communities to bring forward local narratives and perspectives that inform decision-making in ways that are both contextually grounded and equity-focused.

This phase is also an opportunity for researchers to show their respect by genuinely taking steps to understand the community and acknowledging the responsibility that they hold when entering communities. It necessitates a departure from a “them and us” approach and focuses on preparing for a collaborative, reciprocal partnership and co-learning. The essence of this phase is about conducting preparatory “homework” before any community engagement occurs to gain a thorough understanding of the geographical, historical, social, cultural, and religious realities and contexts of each area. It is intrinsically connected to the previous phase and anticipates a research process that is informed and aligned with the community’s values, needs and expectations, a precursor to forming genuine and active relationships.

#### Phase 3: Community aspirations.

The third phase provides an opportunity in the research process for the community to express their aspirations such as hopes, dreams, goals and vision for the future of their own community and recognises diversity within a community [40]. Research approaches often focus on issues and problems through primarily implementing pre-defined solutions, a “cookie-cutter” style approach that may miss the ambitions of the community rather than taking a community strengths-based approach. Therefore, a platform is provided that gives voice to the community and allows for positive, progressive and forward thinking about the future. Engaging with the community at the outset in this manner, it is made clear that the emphasis is on the community itself, encouraging a sense of ownership, empowerment and meaningful engagement.

Co-design and visioning literature emphasise the importance of engaging communities not only in problem identification but also in articulating desired futures [41]. In Aotearoa New Zealand, work on Māori environmental planning emphasises the need to embed iwi (Māori Tribes) and hapū (Māori groups) aspirations to ensure alignment with cultural values and intergenerational wellbeing [40]. Internationally, participatory urban planning methods demonstrate that when aspirations are authentically incorporated, redevelopment is more likely to produce equitable and sustainable outcomes [39]. However, lessons from past projects highlight the risks of tokenism: where aspirations are collected but not acted upon, trust is eroded and communities may disengage [12–15]. To address this, Te Hotonga Hapori framework situates aspirations as central to its cyclical process, ensuring they are not symbolic inputs but are linked to advocacy, action, and re-engagement phases. This approach allows aspirations to be continually revisited, refined, and integrated into redevelopment in ways that reflect collective and culturally grounded visions for the future.

#### Phase 4: Building bridges.

The fourth phase focuses on ensuring dialogue and collaboration between community and the institutions, agencies, and decision-makers responsible for redevelopment. The research team serve as facilitators to building bridges and strengthen the relationship in particular between the community and other stakeholders, especially where trust has been compromised. When community members become discouraged because of misplaced priorities, budgetary constraints and discontinuity of personnel, relationships are compromised or get interrupted, resulting in loss of trust. To counteract the possibility of earlier negative experiences, the research team take steps to facilitate and, if necessary, rebuild the relationships. This means connecting community to relevant stakeholders and being available to support, mediate and advocate for the community along the way as needed. It supports the continuous relationship building and further promotes trust.

This phase reflects literature on cross-sector partnerships, which emphasises that bridging diverse interests is critical for moving from community voice to systemic change [34]. Deliberative democracy scholarship similarly highlights that participatory processes must include mechanisms for negotiation and joint problem-solving if they are to meaningfully influence governance [39]. In the Aotearoa New Zealand context, Te Wahi Hononga research at the interface of Mātauranga Māori and Western science provides both the opportunities and tensions in building bridges across knowledge systems [42]; Lessons from past participatory projects show that without intentional bridge-building, community knowledge risks being sidelined or depoliticised, reinforcing existing power asymmetries [43]. Te Hotonga Hapori framework addresses this by embedding advocacy and accountability into its cyclical structure, creating spaces where community perspectives are actively translated into institutional action.

#### Phase 5: Activation of neighbourhood urban and natural environments.

Once all earlier phases have been successfully initiated, operating in a cyclical and complementary manner, only then is the research team suitably prepared to commence with data collection in the community. In this phase, the focus is on “activation”, meaning identifying elements, features or “hotspots” to enhance spaces and places in the local neighbourhood that improve wellbeing and facilitate a stronger connection with the environment.

This phase draws on literature on placemaking, which demonstrates that community-led actions to activate parks, streets, and public spaces can create a sense of belonging, safety, and wellbeing [44,45]. Tactical urbanism initiatives similarly show that small-scale, low-cost interventions can rapidly demonstrate possibilities for change and mobilise broader support [46]. In the Aotearoa context, Indigenous approaches to environmental stewardship emphasise collective responsibilities to whenua (land) and taiao (natural world), [47] positioning activation as both cultural practice and community development. However, lessons from international examples caution that placemaking can reinforce inequities if not equitably resourced or if benefits accrue disproportionately to more vocal groups [48]. To address these risks, Te Hotonga Hapori framework situates community involvement within a cyclical and relational process, ensuring that activities reflect collective aspirations, are culturally grounded, and are linked to advocacy and structural change rather than remaining symbolic or temporary. The Framework described above will then be enacted through the Community Science Aotearoa Process.

### Community Science Aotearoa process for collecting data and advocacy by communities

**Fig 2:**
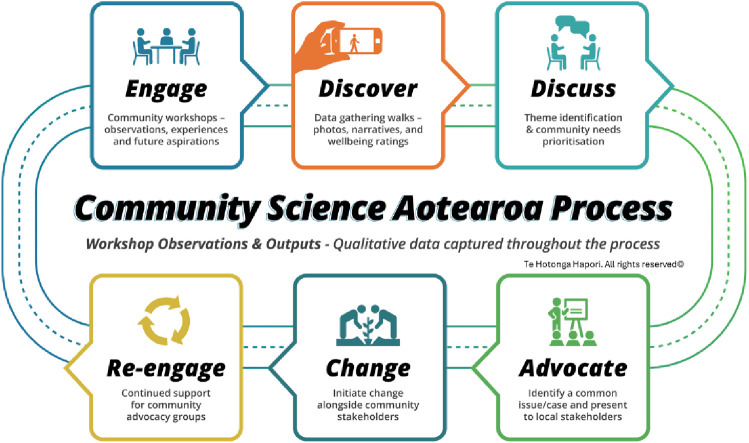
Te Hotonga Hapori’s 6-step Community Science Aotearoa process©, which incorporates and adapts the original evidence-based *Our Voice* Citizen Science for Health Equity method.

Participants will be engaged through an overarching community-based participatory approach called the “Community Science Aotearoa” process. Guided by the Framework and *Our Voice* method, this process involves six steps (Fig 2).

#### Step 1: Engagement.

Engagement reflects longstanding evidence from community-based participatory research (CBPR), which demonstrates that trust, reciprocity, and shared decision-making are foundational for meaningful outcomes [49]. Within Aotearoa, kaupapa Māori research further highlights the centrality of whanaungatanga (relationship building) and mana-enhancing practices as ethical requirements for research partnerships [23]. Lessons from past projects show that without this foundation, later stages of participation risk being perceived as extractive or tokenistic. Participants are invited to participate in a Wānanga or Talanoa (workshop) in their local areas to share their community aspirations in the context of urban development. The session will be recorded, and any audio recordings will be transcribed. To initiate conversations within smaller groups, prompts will be used such as maps of the areas with particular landmarks. Plenary discussions will also be encouraged.

#### Step 2: Discover.

Discovery builds on a wide range of participatory data-gathering methods such as photovoice [50], participatory mapping and GIS [51], and citizen science platforms for health equity [20]. These methods demonstrate that communities are well-positioned to capture fine-grained, locally relevant data that often eludes conventional surveys or technical assessments. At the same time, they caution that data collection must be supported by clear pathways for action; otherwise, communities may disengage if evidence they generate is not acted upon. Participants will undertake a 30-min neighbourhood stroll (walk or wheel), during which they will use the Te Hotonga Hapori smartphone wellbeing application with standard photos and voice capabilities. Participants will be able to take photos and record narratives about places and spaces that enhance or diminish their wellbeing. Examples may include photos of pleasant surroundings, accessible environments, safety, or public green spaces. Participants will also be asked to rate their wellbeing on a scale of −5 to +5 for each photo.

#### Step 3: Discuss.

This step is grounded in traditions of deliberative democracy, collective sense-making, and wānanga. Deliberative sessions emphasise structured dialogue as a way to balance diverse perspectives and negotiate shared priorities [43]. Within Indigenous research contexts, wānanga provide a culturally safe format for in-depth collective discussion that acknowledges historical and cultural realities [52]. Lessons from both literatures emphasise that such dialogue must be well-facilitated and inclusive, otherwise it risks reinforcing the dominance of more vocal groups. Participants are invited to discuss data gathered during their neighbourhood stroll. Facilitated by the research team, participants are supported to discuss photos and voice-recorded thoughts collected in Step 2, identify themes and common topics, prioritise target areas and brainstorm strategies for change, solutions, and actions. Participants will then identify stakeholders and policymakers with whom they would like to advocate for change. At this stage interested participants will form a Community Advocacy Group to further these activities. These discussions and subsequent summaries and conclusions will be recorded, and any audio recordings will be transcribed.

#### Step 4: Advocate.

Step 4 reflects the participatory turn in governance, where communities mobilise evidence to influence policy and decision-making [53]. The Our Voice citizen science model has shown how resident-collected data can be used to successfully advocate for environmental and policy changes that promote health equity [31]. At the same time, lessons from urban redevelopment highlight that advocacy must be coupled with institutional accountability, as symbolic consultation without systemic responsiveness can erode trust and legitimacy [54].

Participants will form a Community Advocacy Group for their areas with the support of the research team, prepare a case for change and identify potential solutions. Any information relating to the case such as literature, reports, and data will be gathered and utilised to make the case. Stakeholders and policymakers will be identified and invited to the advocacy session. Before the case is finalised, feedback will be sought from the Community Advocacy Group. All discussions and decisions will be recorded. Any audio recordings or feedback will be transcribed.

#### Step 5: Change.

This step connects directly to literatures on co-design, tactical urbanism, and participatory planning, which demonstrate how small-scale interventions can catalyse broader transformation [55]. However, critiques of regeneration projects caution that unless equity considerations are prioritised, such changes may contribute to gentrification or exclusion, undermining community wellbeing [56].

The Community Advocacy Group, with the support of the research team, will present their case and findings to the stakeholders and policymakers and discuss potential strategies, initiatives and solutions. The research team will facilitate these relationships and meetings. An agreed Action Plan and communications structure will be formed and finalised from these meetings.

#### Step 6: Re-engagement.

The last step is perhaps the most distinctive step of Community Science Aotearoa process, embedding an explicit cycle of return and continuity. This responds to critiques that many participatory projects end once data are collected or advocacy is attempted, leaving communities feeling abandoned [57]. Iterative re-engagement reflects Māori principles of utu (reciprocity) and ongoing whanaungatanga, [23,33] and has been identified in CBPR as a predictor of long-term trust and sustainability [32]. By institutionalising this return phase, the process ensures that communities remain central and that relationships are maintained over time

The research team will re-engage with the Community Advocacy Group at different times to support and monitor progress. The research team will support future meetings and linking the group with stakeholders where needed. An outcome and process evaluation will also be conducted to determine the level of ongoing engagement and actions between a self-supporting community and cross-sector stakeholders and policymakers.

### Stakeholder workshop

At the completion of data collection and following preliminary analysis of the qualitative data, stakeholders will be invited to a workshop to reflect on and discuss the results. The stakeholders will be those who were officially involved with the redevelopment areas. The purpose of the workshop will be to provide a safe environment for the stakeholders to interact, share their insights and lessons learnt for future redevelopment processes particularly focusing on ways to maintain residents’ wellbeing. We will explore short- and long-term opportunities and solutions from the perspective of the stakeholders.

### Development and rationale of the Te Hotonga Hapori Engagement Framework and Community Science Aotearoa process

#### Positioning within existing approaches.

While a variety of participatory approaches have been employed in urban development contexts, there remains limited empirical evidence on what approaches are most effective [58]. Calls for decolonising participatory design emphasise the need to move beyond systematic over-study of marginalised groups and the limited real-world impact of much participatory research [23,59]. Widely adopted international participatory designs have often struggled to meet community needs in practice [60]. At the same time, scholars have highlighted the importance of shifting practice from imperialistic methodologies toward approaches that centre empowerment, and co-creation, while recognising the challenges these transitions entail [61]. The Our Voice Citizen Science for Health Equity method (Our Voice) [19] that involves participants in the research process from the start is an approach inextricably linked to empowerment. The Our Voice method was chosen as a starting point because it is an internationally recognised and empirically validated approach for connecting community lived experiences with policy and environmental change. Unlike many participatory research methods that focus primarily on data collection or consultation, Our Voice provides a structured yet flexible process that enables participants to identify priorities, document issues in their environments, and engage in advocacy with decision-makers. It has been successfully implemented in diverse urban and health contexts globally, demonstrating its potential to influence local planning and health equity outcomes [20,30,31,62–68]. This made it an appropriate foundation for our study, which is similarly focused on embedding community perspectives into urban redevelopment. When combined with Community-Based Participatory Research (CBPR), it has the potential to deliver direct and meaningful impacts on society and social infrastructure [69]. By integrating citizen science with a participatory action approach and Citizen Science **by** the people [19], it empowers participants to provide signficant and meaningful local information about their environment, prioritise concerns, interprate data through their lived experiences and engage in cross-sector dialogue to generate tangible and lasting improvements within their neighborhoods. In the New Zealand–Aotearoa context, the development of a framework tailored to local cultural and historical realities was particularly needed, to give contemporary expression to the Treaty partnership and to embed Indigenous perspectives in urban development processes [70]. Grounded in Māori principles and responsive to the Aotearoa New Zealand context, Te Hotonga Hapori Engagement Framework provides an iterative foundation for meaningful community engagement. It emphasises co-production of knowledge, yet it extends this through embedding mana (uplifting)-enhancing practices and cyclical re-engagement stages. While aligned with existing participatory traditions, the framework charts a novel path by systematically combining citizen science methodologies with Indigenous perspectives in a way that responds to both local cultural contexts and structural challenges in urban redevelopment.

### Framework and process development

Te Hotonga Hapori Engagement framework and process were co-developed through a series of six collaborative sessions held over 12 months with representatives from health promotion agencies serving Māori and Pacific communities in Auckland. These sessions were guided by culturally informed processes and sought to understand community priorities, cultural contexts, and daily realities. Insights were iteratively integrated into the framework and process through discussion, feedback, and refinement. The Process drew on the *Our Voice* method [19] combined with CBPR principles. The community cultural advisors were deliberately centred in this stage of co-development, while institutional and developer perspectives were not included, to prioritise community voices in shaping the foundation of the framework.

### Analytical approach and findings

Content analysis of session insights involved coding, identifying recurrent themes, and grouping data into categories that informed framework components. Cross-validation with participants ensured accuracy, relevance, and cultural sensitivity. Findings highlighted the need for a framework grounded in Te Tiriti o Waitangi principles – Whanaungatanga (relationship-building), Kāwanatanga (shared governance), Tino Rangatiratanga (self-determination), Ōritetanga (equity), and Wairuatanga (spiritual and cultural respect). Participants emphasised iterative engagement before, during, and after projects to sustain trust, the prioritisation of collective wellbeing, and the importance of cultural alignment. As a consequence the process resulted in six iterative steps: (1) Engage, (2) Discover, (3) Discuss, (4) Advocate, (5) Change, and (6) Re-engage. These steps are not intended as a linear sequence but as a cyclical process in which communities and stakeholders can re-enter at multiple points. It reflects the ongoing and relational nature of engagement, ensuring that community voices are not only heard once but continually revisited as redevelopment progresses. While the *Our Voice* method was valued, participants expressed discomfort with the colonial connotations of the term *“citizen”*, preferring *“community”* to reflect collective engagement, while affirming the value of *“science”* in legitimising local knowledge and practice. Consequently, the Process was named Community Science Aotearoa process.

### Theoretical and empirical basis for effectiveness

The framework is expected to be effective because its design elements are supported by a growing body of evidence. Iterative engagement processes have been shown to strengthen trust and lead to more sustainable outcomes in community–institution partnerships [32]. The integration of citizen science approaches, particularly when combined with community-based participatory research, has been found to increase local relevance of data and enhance policy uptake [30]. Embedding Māori principles and Pacific perspectives aligns with literature emphasising that culturally grounded methods are critical for Indigenous community participation and wellbeing [23,33]. Moreover, the cyclical structure of Te Hotonga Hapori addresses critiques of participatory methods as “one-off” or extractive, by ensuring re-engagement and continuity, which are identified as key to sustaining community trust and impact [57,71]. Taken together, these elements suggest that the framework and process are culturally appropriate and also theoretically and empirically supported as a robust approach for advancing wellbeing and lived experience in urban development contexts.

### Minimising risk of participant burden and less confident voices

Participatory approaches may risk imposing additional burden to communities. To minimise this risk, engagement processes need to be culturally grounded, reciprocal, and guided by values [23,33,72]. Te Hotonga Hapori Engagement Framework is driven by Māori and Pacific principles and values, is designed to redistribute responsibility by ensuring that stakeholders and decision-makers are accountable for responding to community-identified priorities, rather than requiring communities to continually demonstrate the importance of their lived experiences.

In addition, we embedded safeguards to protect wellbeing throughout. Engagement activities will be kept short, facilitated in culturally appropriate ways, and scheduled flexibly to accommodate participants’ commitments. Reciprocity is built into the process, including provision of koha (appreciation gift), and food, as well as returning findings in culturally relevant and useful formats. Continuous consent will be emphasised, ensuring participants may step back or re-join as desired. Importantly, mechanisms will be in place to ensure participants see how their contributions are acted upon, reducing fatigue or disillusionment. These safeguards reflect lessons from community-based participatory research, which emphasises trust and shared benefit as critical to sustaining wellbeing [73,74], and from critiques of tokenistic engagement, which show that excessive demands without tangible outcomes undermine community trust [75].

To ensure inclusion of less vocal participants, engagement will be complemented by small group facilitated discussions, with multiple options for contributing (oral, written, and visual) and opportunities for anonymous input. Expert facilitators will be involved using tested approaches to encourage participation from all community members, particularly those less likely to speak in any forums. Facilitators will ensure a positive and respectful environment from the outset using a variety of techniques (e.g., participant created terms of reference).

### Te Hotonga Hapori app

Te Hotonga Hapori app is a custom-designed, multi-platform smartphone application created to assess wellbeing through user engagement across various projects. It was developed to track participants’ wellbeing by collecting daily responses over a designated period. The app also allows participants to capture photos, record narratives, and document their subjective wellbeing in relation to the photos taken. Te Hotonga Hapori app was developed in collaboration with Inserm, Paris (National Institute of Health and Medical Research). It was based on an existing app, the Eco-Emo tracker, [76] initially used to track participant location in real time. Our programme added user-friendly ecological momentary assessment (EMA) with image/audio recording functionality to allow the simultaneous collection of location with real-time survey data. It continues to exist as a joint partnership by Inserm and AUT.

All data generated through the app will be managed in accordance with approved ethical protocols, including secure storage, de-identification of personal information, and restricted access to authorised researchers only. Data sovereignty considerations are central: communities will retain agency over how data are used, and findings will be fed back in culturally appropriate formats. Privacy and consent processes are embedded at each stage of participation.

### Data analysis

Descriptive statistics will be produced in relation to the number and typology of geocoded photos and narratives collected. Qualitative thematic analysis will be used for narrative data obtained from downloaded app data, and community and group sessions. We will seek to understand from the data the context and experiences of participating residents in their surrounding neighbourhood environments and how it impacts their wellbeing, along with stakeholder insights. Data from all six steps of the Community Science Aotearoa process will be analysed, including input from community workshops, neighbourhood walks, advocacy group sessions, stakeholder workshops, and outcomes from the advocacy process. This may include meeting transcripts, photos, audio-recordings, minutes from board meetings, reports, policy changes, budget allocations, and stakeholder engagement levels.

Braun and Clarke’s Thematic Analysis Framework [77,78] will guide researchers through an iterative process consisting of six steps (detailed below) of data analysis, which include (1) *Reviewing data* to engage deeply with and ask questions of the data – noting and recording differences, similarities, thoughts and situations of significance as well as recognising the analyst’s own responses to the data; (2) the *Coding* of data focusing on the research question; (3) *Thematic development* using the Thematic Networks Analytic Tool from Attride-Stirling [79]; (4) *Potential themes* and subthemes review, and development and examination of a thematic map; (5) *Naming and defining themes* and identifying thematic relationships; and (6) *Locating exemplars*, the final analytic step where the themes are clearly defined and written in a way that demonstrates congruence with the research question.

### Ethics and dissemination

The study has received approval from Auckland University of Technology Ethics Committee (AUTEC 22/29) in accordance with the Royal Society Te Apārangi’s Code of Professional Standards and Ethics in Science, Technology and the Humanities 2019 (the Royal Society NZ Code), and Kāinga ora-Homes and Communities (HPREC-045–23). Residents (aged 18+) in the four areas of Waikōwhai, Wesley, Oranga and Aorere who completed an earlier project (AUTEC approval number 21/361) and consented to being contacted for this project will be eligible and will be invited to participate. An invitational letter will be sent out with the participant information sheet. Participants will be invited to a community meeting with AUT researchers and community leaders to explain the project. The AUT researchers, community leaders and the participants will be working together closely from the moment the participants agree to participate in the study. Utilising the Community Science Aotearoa process will ensure close collaboration and co-creation.

### Community-shaped engagement, reciprocity, and recruitment

Prior to any contact with resident participants, the research team will meet with community leaders to discuss the project. Māori and Pacific peoples input will be sought on the values underpinning the research, details of the study, outcomes to be investigated and the protocols for engagement, data collection and data management. Input will also be provided by research partners and community stakeholders. A community advisory network will be established to enable ongoing input throughout the research, data interpretation of data, and mobilisation of knowledge.

Guided by Te Hotonga Hapori Engagement Framework and Community Science Aotearoa process, community engagement is therefore intentionally designed as a reciprocal and adaptive process. Rather than prescribing a fixed sequence of activities, the framework allows communities to shape how engagement unfolds, with hui (meetings) and wānanga providing spaces for collective dialogue, negotiation, and adaptation at each stage. Reciprocity (utu) is embedded through practices such as koha, provision of food, and the return of findings in accessible and culturally appropriate forms, ensuring that communities experience tangible benefits throughout the process. Engagement will be iterative and cyclical, with opportunities for feedback and re-engagement at every stage, reflecting the back-and-forth dynamics of authentic relationship building.

### Research team and implementation

The research team is central to the implementation of Te Hotonga Hapori Engagement Framework and Community Science Aotearoa process and is not positioned as objective observers. The team comprises academic investigators with expertise in urban health, urban design, wellbeing, participatory methods, and Indigenous research, alongside Māori and Pacific cultural advisors, health agency partners, and technical developers. Each member contributes specific expertise, from facilitating sessions and supporting community engagement, to ensuring cultural protocols are upheld, and functionality of the Te Hotonga Hapori app. The team’s role is to create culturally safe spaces, provide facilitation that is mana-enhancing, and support participants in every step of the process including the use the app and other engagement tools. The research team will also synthesise findings through iterative dialogue with communities, ensuring accuracy and cultural relevance, and will take responsibility for translating insights into formats that can be used by decision-makers. In this way, the research team will play an active and accountable role in supporting authentic engagement and ensuring that the process delivers both community benefit and actionable outcomes.

## Discussion

This study presents Te Hotonga Hapori Engagement Framework and the Community Science Aotearoa process as a novel, culturally grounded approach to participatory engagement in urban redevelopment. While many participatory methods are effective at identifying local issues, they often fail to ensure sustained engagement or to translate insights into structural change. The approach presented here addresses these gaps by combining international experience with the *Our Voice Citizen Science for Health Equity* method (*Our Voice*) [68] and adapting it through Māori and Pacific cultural principles. Te Hotonga Hapori Engagement Framework provides guiding phases that embed relationship building, recognition of historical and cultural realities, and the activation of neighbourhood environments. Community Science Aotearoa operationalises these principles through a six-step cyclical process of engagement, discovery, discussion, advocacy, change, and re-engagement. Together, they provide a structured but flexible protocol that places community voices at the centre of redevelopment decision-making. Both the Framework and the Process can potentially be adapted for other populations when employing community and citizen science methodologies interested on ensuring cultural relevance. This adaptation can ensure greater equity across diverse cultural groups through authentic and culturally-relevant engagement [80,81].

The framework and process make three contributions to the participatory research field. First, they explicitly embed Indigenous and Pacific cultural principles within an established international method, demonstrating how global approaches can be adapted for local contexts. Second, they incorporate reciprocity and institutional accountability as core features, addressing common critiques of participatory approaches as tokenistic or extractive. Third, the framework and process are cyclical rather than linear, institutionalising the return to communities and thus helping to sustain trust, continuity, and shared responsibility for change.

Te Hotonga Hapori Engagement Framework sits within a broader tradition of participatory and decolonial approaches to community engagement, such as CBPR [82], indigenous-led frameworks in urban development [47] and citizen science methods adapted for planning and health contexts [20,62,66,68]. Drawing from these approaches, it seeks to democratise knowledge production and strengthen community agency, but it extends them through embedding Māori principles and a cyclical process of re-engagement that ensures continuity of relationships beyond initial project outcomes. The framework was not developed in isolation: its design was shaped through iterative hui with representatives from health and social agencies. These engagements consistently emphasised the importance of relational trust, recognition of cultural and historical narratives, and the need for practical mechanisms enabling communities to re-engage after advocacy efforts. Such insights informed the development of the Engage–Discover–Discuss–Advocate–Change–Re-engage cycle of the Community Science Aotearoa process guided by the Framework. While aligned with existing participatory traditions, the framework charts a novel path by systematically combining citizen science methodologies with Indigenous perspectives in a way that responds to both local cultural contexts and structural challenges in urban redevelopment.

Citizen science was selected as a methodological foundation because it enables communities to actively participate in the identification, interpretation, and advocacy of issues that directly affect their lived environments. A growing body of literature demonstrates that citizen science can democratise data collection, enhance local relevance, and catalyse changes in urban and health policy [18,30]. The Community Science Aotearoa process builds on the internationally recognised *Our Voice Citizen Science for Health Equity* method [20], which has shown effectiveness in supporting communities to identify local priorities and influence policy and practice [20,30,31,62–68]. What is unique about the Community Science Aotearoa process is its integration of Māori and Pacific values and its explicitly cyclical design. Whereas many citizen science initiatives stop at issue identification, this process embeds relational accountability and advocacy, requiring institutions to respond to community-identified priorities. Its cyclical stages—Engage, Discover, Discuss, Advocate, Change, and Re-engage—go beyond data generation to support the translation of community insights into practical interventions in the urban environment, while maintaining sustained relationships and community empowerment.

The advantage of using the Community Science Aotearoa process is that it goes beyond issue identification and enables translation of scientific evidence into actions for practical application. For example, connecting community members with relevant stakeholders to present community-generated evidence can facilitate informed decision making for the benefit of the local communities. For that reason, we have incorporated in the process a reflective step where stakeholders come together to discuss the evidence generated by the community so that current and future redevelopments are informed, but also to capitalise on the opportunity for enhanced collaboration. There is the potential for a paradigm shift where communities take a central role during urban redevelopment. We see promise in this process where local knowledge and experiences, and community priorities are leveraged to create sustainable and equitable standards for urban development.

While citizen science, community science, participatory research, photovoice and other approaches in health, wellbeing and environmental justice research [25,26] have been utilised within communities, it has not reached a significant scale to inform actions [83,84]. In the housing and development areas to date, we know of only one example where the Scottish Government (Riaghaltas na h-Alba) requires by law, through the Housing (Scotland) Act 2001, for landlords to have a tenant participation strategy [85], and produce a guide for successful tenant participation [86]. When such participatory processes are not part of the parcel, it can significantly affect the full understanding of community needs and wants, inhibiting the development of inclusive solutions [12–14].

CBPR and related traditions emphasise trust and relationship building as prerequisites for participation, yet sustaining engagement over time remains a persistent challenge. By embedding a formal *re-engagement* phase, Community Science Aotearoa advances the literature on sustaining participatory processes. It also contributes to theoretical debates about the role of lived experience as expertise, recognising that community knowledge is not supplementary but essential for shaping interventions that are both effective and equitable.

For urban redevelopment practice, the framework provides a practical pathway for embedding community voices in contexts where redevelopment often prioritises technical expertise and market priorities. Lessons from past projects such as Pruitt-Igoe, the Heygate Estate, and HOPE VI highlight the risks of displacing residents, undermining trust, and eroding community identity when participation is symbolic or absent. Te Hotonga Hapori responds directly to these lessons by situating redevelopment in historical and cultural realities, requiring cross-sector bridge-building, and embedding accountability mechanisms that extend beyond the project team to include decision-makers and institutions. The protocol is thus relevant not only for researchers but also for government agencies, housing providers, and councils seeking to implement equitable and sustainable redevelopment.

For Māori and Pacific health equity, the framework demonstrates how culturally grounded approaches can enhance both relevance and legitimacy. Incorporating Māori principles such as whanaungatanga (relationship-building), manaakitanga (care and reciprocity), and tino rangatiratanga (self-determination) ensures that processes are not only procedurally inclusive but also aligned with Indigenous values and aspirations. Similarly, Pacific perspectives on collective wellbeing and interdependence inform the design of engagement processes that move beyond the individual to emphasise whānau and collective benefit. In this way, the framework contributes to the advancement of Vision Mātauranga principles and responds to longstanding calls for research approaches that are both culturally safe and practically impactful.

Understanding the complexity and impacts of urban redevelopment calls for a “mosaic” of evidence [87,88]. Stakeholders and decision-makers for urban design and planning who seek to improve urban environments, welcome evidence that is robust and relevant to that context [89]. Local knowledge and lived experiences can help fill those evidence gaps [90], contributing towards the improvement of the external and internal validity of a body of evidence [87]. Citizen and Community Science methods can support communities to advocate for effective urban redevelopment projects through evidence-based and systematic data gathering, that is context specific.

The findings generated through Te Hotonga Hapori-Community Wellbeing and Lived Experiences project should be understood as one component of a broader mosaic of evidence. While they will provide in-depth insights into lived experiences and cultural priorities, they will be most powerful when interpreted alongside other data sources, such as population health indicators, built environment measures, and policy analyses. This dialogue between diverse forms of evidence ensures that community knowledge is not siloed, but rather contributes directly to the planning, design, and implementation of urban redevelopment. The merits of this mosaic approach include enhanced relevance, responsiveness, and accountability; limitations include the challenges of integrating heterogeneous data sources and navigating tensions between local priorities and system-level constraints. Nevertheless, this pluralistic approach offers a more holistic and equitable basis for decision-making in urban environments.

At a global level, the study protocol illustrates how international participatory methods can be localised to different cultural and institutional contexts. Adaptation of *Our Voice* to the Aotearoa setting demonstrates the importance of contextual flexibility and provides a model for other countries seeking to align citizen science with Indigenous and community worldviews. By situating the framework within a broader “mosaic of evidence,” the approach also shows how community-generated insights can be placed in dialogue with epidemiological, policy, and planning data to create richer, more responsive bases for decision-making.

The findings of the current study will have wider applications for redevelopment practitioners in three ways. Firstly, the results from this project will contribute to the ‘mosaic’ of existing evidence by capturing rich information on how residents from under-resourced communities may experience neighbourhood urban form and why those experiences affect their wellbeing. Secondly, we are conscious of the need to gather robust evidence that can be directly applied to urban redevelopment management. Thirdly, by wrapping a robust science framework around the engagement process, we continually learn and can generate evidence on what works in translating research into practice.

There are several strengths to this study protocol. One of the key strengths is the strong emphasis on community being at its centre to ensure that the needs, concerns, and lived experiences are clearly communicated so that decisions around redevelopment, including through the disruptive construction phases are fit-for-purpose and genuinely enhance the wellbeing of residents. The use of Te Hotonga Hapori Engagement Framework to guide data collection, is informed by indigenous perspectives and therefore culturally responsive and acknowledges the importance of cultural identity, relationships, and the physical environment in shaping wellbeing. It also promotes inclusivity and respect for indigenous knowledge, which is often missed in urban redevelopment processes. The Community Science Aotearoa process serves two purposes: to gather valuable data and empower residents with the tools and knowledge to advocate for their communities. It is hoped that this capacity-building aspect of the study can lead to sustained community engagement and advocacy beyond the duration of the project (i.e., ripple effects). Such ripple effects have been reported in a number of other *Our Voice* projects [30,31].

There also limitations to the study protocol. There may be challenges in ensuring that the less vocal residents are heard. It is possible that past experiences leading to mistrust and exclusion or other factors that may prevent participation, may result in some residents being less inclined to participate which could lead to unbalanced representation of community needs, priorities and aspirations. However, we will address this by engaging alternative methods to elevate quieter voices by offering small group facilitated discussion and multiple modes of contribution (visual, oral, written) with expert facilitators running the sessions. In addition, during recruitment, we will seek the support of local trusted community leaders and organisations. We will also accept any invitations and attend community events to discuss the research and distribute information with meaningful content for better reach. We also anticipate that some expectations around redevelopment may be unrealistic given budget, time and resource constraints. We will formulate a protocol on how to communicate clearly the scope of the project and what the project can realistically achieve. We plan to use prioritisation activities to support realistic and achievable expectations and provide a forum where the residents can build consensus. It should not be underestimated that while the participatory nature of this study is its strength it could also be a limitation as it can be time-consuming and resource intensive. To ameliorate such potential limitations, key expert personnel will be assigned to the study who will dedicate their time to ensure that nothing is overlooked, and that the community receives full support. In addition, the study will be implemented in phases and each community will be focused on separately. Also, we will work closely with our partners and seek their support during the implementation process. While a main strength of this study is its cultural responsiveness, it may also be a limitation in generalising the findings to other contexts. Therefore, we will document decision-making processes and actions taken throughout each phase to help other researchers adapt the approach to their contexts. We will also share lessons learned, including both successes and challenges, to enable replication or adaptation. There also may be uncertainty in terms of the longer-term impacts of the study, especially once the project is finished. To address this, we will be supporting communities to form advocacy groups. We have included a step in the Community Science Aotearoa process of Re-engagement to ensure check ins occur and document any ripple effects. Finally, the study is only available in the four designated areas. However, funding will be sought in the future to extend the research and its insights to other areas.

In essence this research will offer a paradigm shift where communities are given the platform to collect their own data and are empowered as key participants to inform the redevelopment planning and implementation process. Communities working together with relevant stakeholders may enhance the chances of implementing solutions that are fit-for-purpose.

### Strengths and limitations of this study

1. The study is guided by Te Hotonga Hapori Engagement Framework, which is a culturally informed and context-specific framework, specifically developed to guide the research.
2. Community Science Aotearoa process will be applied, adapted from the evidence-based and internationally applied *Our Voice* Citizen Science for Health Equity Research method.
3. The project is occurring within Te Hotonga Hapori – Connecting Communities research programme designed to ensure that wellbeing of the residents during redevelopment is addressed in a holistic manner.
4. The study is limited to the four areas of urban redevelopment occurring in Auckland, New Zealand.

## Supporting information

S1 Inclusivity in global research-questionnaire final. (DOCX)

## Terms

Te Hotonga Hapori – Connecting Communities was gifted to the research team by Dr Tā (Sir) Tīmoti S Kāretu KNZM QSO, one of Māoridom’s most respected academics and linguists in 2020, on request of Professor Tania Ka’ai.

The non-English words in the manuscript are in the Māori language. In Māori, ‘Te’ is equivalent to ‘the’ in English, and therefore, when used in an English sentence, it is often omitted to avoid redundancy.
