# Supplementary material for: Community wellbeing and lived experiences during urban redevelopment in New Zealand: Te Hotonga Hapori – Connecting communities qualitative study protocol
Source: PLoS One. 2025 Oct 3;20(10):e0333480. doi: 10.1371/journal.pone.0333480 (PMC12494257; doi:10.1371/journal.pone.0333480)
Supplement: S1 — (DOCX) [file pone.0333480.s001.docx]

Inclusivity in global research

PLOS’ policy on inclusivity in global research aims to improve transparency in the reporting of research performed outside of researchers’ own country or community and ensures that PLOS publications reporting global research adhere to high standards for research ethics and authorship. Authors of relevant research articles may be asked to complete the questionnaire below, which outlines ethical, cultural, and scientific considerations specific to inclusivity in global research. This questionnaire may be requested when researchers have travelled to a different country to conduct research, if research uses samples collected in another country, research with Indigenous populations or their lands, or if research is on cultural artefacts. Researchers travelling to another country solely to use laboratory equipment will not normally be required to complete the questionnaire. However, the questionnaire can be requested at the journal’s discretion for any submission – if you have been requested to complete this questionnaire by the PLOS journal you submitted to, please do so.

Please complete the questionnaire below and include this as a Supporting Information file with your manuscript. Note that if your paper is accepted for publication, this checklist will be published with your article in the supporting information files. Please ensure that you reference the checklist in the main body of your manuscript. We suggest adding a subsection ‘Inclusivity in global research’ to your Methods section and adding the following sentence: “Additional information regarding the ethical, cultural, and scientific considerations specific to inclusivity in global research is included in the Supporting Information (SX Checklist)”

The questions have been designed to be applicable to a wide range of study types, and there are subsections for both human subjects research and non-human subjects research. If any of the questions are not relevant to your research please mark them as “N/A” as appropriate.

**Ethical considerations, permits and authorship**

*This section is applicable to all research types.*

Provide details as to who granted permissions and/or consent for the study to take place in the Methods section of your manuscript. This should include the names of **all** ethics boards, governmental organizations, community leaders or other bodies that provided approval for the study. If individuals provided approval refer to these people by their role or title but do not list their name(s).

Reported on page number: 11

*The study has received approval from Auckland University of Technology Ethics Committee (AUTEC 22/29) in accordance with the Royal Society Te Apārangi’s Code of Professional Standards and Ethics in Science, Technology and the Humanities 2019 (the Royal Society NZ Code), and Kāinga ora-Homes and Communities (HPREC-045-23).*

If there were any deviations from the study protocol after approval was obtained please provide details of these changes in the Methods section of your manuscript.
Did this study involve local collaborators that are residents of the country where the research was conducted or members of the community studied? If you do not have any authors from said communities, please provide an explanation for this below.

*Yes. This study was developed in Aotearoa New Zealand, where the research team is based. The Te Hotonga Hapori Engagement Framework was co-developed through collaborative sessions with Māori and Pacific health promotion agencies, ensuring that the perspectives of communities central to the study are embedded from the outset. While this protocol paper is authored by the academic team, Māori and Pacific advisors and agency partners were actively involved in shaping the framework and will continue to be integral collaborators in the implementation and future dissemination phases. We are committed to inclusive authorship practices and anticipate that community and Indigenous collaborators will be co-authors on subsequent papers reporting study findings.*

Reported on page number: No deviations.

Everyone listed as an author should meet PLOS’ criteria for authorship and all individuals who meet these criteria should be included in the author byline, rather than the acknowledgements. For further information please see the journal’s Authorship Policy.

**Human subjects research (e.g. health research, medical research, cross-cultural psychology)**

Did you obtain written informed consent from a representative of the local community or region before the research took place? How did you establish who speaks for the community? Details of written informed consent obtained from study participants should be reported separately in the Methods section of your manuscript.

*As this manuscript describes a study protocol, no community-level written consent was required prior to publication. The development of the Te Hotonga Hapori Engagement Framework involved collaborative sessions with Māori and Pacific health promotion agency representatives, who provided input on cultural perspectives and community priorities. In Aotearoa New Zealand, authority is not vested in a single person to speak for a community; instead, engagement is conducted through recognised leaders, iwi, hapū, whānau, and agency representatives, following Māori principles and Vision Mātauranga guidance. For subsequent phases of the study, we will obtain ethics approval and written informed consent from individual participants, as well as ensure that engagement with local leadership structures and governance processes is undertaken in a culturally appropriate and mana-enhancing way.*

How did members of the local community provide input on the aims of the research investigation, its methodology, and its anticipated outcome(s)?

*The aims, methodology, and anticipated outcomes of this study were informed through a series of collaborative sessions with Māori and Pacific health promotion agency representatives. These sessions provided space for discussion of community priorities, cultural values, and lived realities, which shaped the design of the Te Hotonga Hapori Engagement Framework and its operationalisation through the Community Science Aotearoa process. Community input emphasised the need for an approach that is culturally grounded, iterative, and focused on collective wellbeing. Anticipated outcomes, including strengthened community voice in redevelopment, improved equity, and the translation of evidence into practice, were identified as priorities by these local collaborators and were embedded into the study design from the outset.*

When engaging with the local community, how did you ensure that the informed consent documents and other materials could be understood by local stakeholders?

During framework development, engagement was conducted in English. Technical terms were explained, and discussions were guided by principles of whakawhanaungatanga (relationship building) to create safe spaces for questions and clarification. For subsequent phases involving research participants, all consent documents and study materials will be reviewed in partnership with cultural advisors to ensure they are written in accessible language and translated where needed. This process is designed to ensure that documents are understandable, culturally relevant, and respectful of the communities involved.

Will the findings of the research be made available in an understandable format to stakeholders in the community where the study was conducted (e.g. via a presentation, summary report, copies of publications, etc.)? Please provide details of how this will be achieved.

*Yes. Findings will be returned to participating communities in multiple accessible formats. These will include plain-language summary reports, presentations delivered in community settings, and digital materials co-designed with local partners (e.g., infographics, short videos). In addition, where appropriate, hui and fono will be held with Māori and Pacific stakeholders to discuss findings kanohi ki te kanohi (face-to-face) and to ensure they are interpreted in ways that are culturally meaningful. Copies of open-access publications will also be shared directly with agency partners and made available to community members. These dissemination strategies were identified during framework development as important to maintaining trust, reciprocity, and ongoing collaboration.*

**Non-human subjects research using specimens/ animals collected as part of the study, or those housed in archival collections. Examples include archaeology, paleontology, botany and zoology.**

Did the permission you obtained from a local authority to perform the study include an agreement on access to outputs and benefit sharing? This may include procedures to enable fair distribution of the benefits and resources arising from the research performed. Please include any details of Prior Informed Consent and Benefit Sharing Agreements obtained. These may be required by field-specific regulations, for example the Convention on Biological Diversity (CBD) and the associated Nagoya Protocol.

NA

If the material used in your study was imported, please A) provide the year it was imported and B) indicate whether permits were obtained to import/export the materials used, C) provide details of any permits obtained. If this information is not available, please indicate this.

NA

If you used archival specimens, please state how the material used in your study was acquired by the institute it is held in and provide details of any permits obtained for the original excavations/ sample collection. If this information is not available, please indicate this.

NA

How was the potential cultural significance of the materials collected in your study to local communities considered in your research design? Were Indigenous peoples and/or local researchers and institutions involved with archaeological excavations / collection of specimens? If so, please provide a description of their involvement.

NA

If your manuscript includes photographs of human remains please indicate whether authors obtained permission from descendants or affiliated cultural communities to do so.

NA
